# The effect of different dosage of intranasal dexmedetomidine on preventing emergence delirium or agitation in children: A network meta-analysis of randomized controlled trials

**DOI:** 10.1371/journal.pone.0304796

**Published:** 2024-09-06

**Authors:** Yizheng Li, Yi Jiang, Liangcheng Zhang

**Affiliations:** 1 Department of Anesthesiology, Fujian Medical University Union Hospital, Fuzhou, China; 2 Department of Anesthesiology, Wenzhou Hospital of Integrated Traditional Chinese and Western Medicine, Wenzhou, China; Sapienza University of Rome: Universita degli Studi di Roma La Sapienza, ITALY

## Abstract

**Background:**

The clinical evidence for the effects of different doses of intranasal dexmedetomidine on emergence delirium/ emergence agitation (ED/EA) in children is lacking.

**Methods:**

We searched the PubMed, EMBASE and Cochrane Library from the establishment of the databases until December 30, 2023. All randomized controlled trials that evaluated the effect of different dosage of intranasl dexamedetomidine in children younger than 18 years on postoperative ED/ EA were included. Data analysis was conducted using R 4.3.0.

**Results:**

A total of 15 randomized controlled trials involving 1566 children were included. Compared to 0.5 μg/kg (RR = 4.81, 95%CI = 1.66–13.94), and normal saline (RR = 8.23, 95%CI = 4.63–14.65), intranasal dexmedetomidine at doses of 2 μg/kg significantly reduced the incidence of ED/ EA in children. 2 μg/kg was the most effective dosage in reducing the incidence of ED/ EA (Probability of rank = 0.75), the incidence of severe ED/ EA (Probability of rank = 0.45), and ED/ EA score (Probability of rank = 0.65). Moreover, intranasal dexmedetomidine at doses of 2 μg/kg significantly reduced the PACU pain compared to 0.5 μg/kg (RR = 0.42, 95%CI = -0.22–1.06), 1 μg/kg (RR = 0.18, 95%CI = -0.26–0.63), 1.5 μg/kg (RR = 1.00, 95%CI = -0.54–0.75), and normal saline (RR = 8.23, 95%CI = 4.63–14.65), with a probability of rank = 0.45.

**Conclusion:**

2μg/kg intranasal dexmedetomidine is the optimum dose for reducing the occurrence of ED/ EA and postoperative pain. However, further research is required to verify our findings.

## Introduction

Emergence delirium or emergence agitation (ED/ EA) are common postoperative complications in children that characterized by behaviors such as crying, fear, restlessness, and disorientation occurring in the early stages during anesthesia recovery period according to diagnostic criteria of the Pediatric Anesthesia Emergence Delirium (PAED) scale [1]. Meta-analysis by Farag et al. [2] showed the incidence of ED/ EA was 31.4% in pediatric patients underwent sevoflurane anesthesia and surgery. ED/EA in children led to serious consequences such as vomiting, aspiration, laryngospasm, and notably extended hospital stays [3, 4]. Therefore, effective interventions to reduce ED/ EA in pediatrc patients is an issues to be solved.

Dexmedetomidine (DEX), a highly selective α2-adrenoceptor agonist, was a promising medication in this context [5]. Until now, intranasal DEX has been widely used in pediatric patients and showed effectively in reducing the incidence of ED/ EA [6, 7]. Li et al. [8] indicated that children under 3 years, DEX takes sedative and analgesic effect in 20 minutes, maintains a two hour time, and has less effect on systemic vascular and respiratory depression, moreover, intranasal DEX would not induce nasal irritation, making it a suitable option for preoperative medication. However, the dosage of intranasal DEX differed among studies, and the optimal dose for preventing postoperative ED/ EA remains inconclusive.

This network meta-analysis aimed to investigate the optimal dose for preventing postoperative ED/ EA in children, and provided clinical medication guidance.

## Methods

This study was conducted according to the Preferred Reporting Items for Systematic Reviews and Meta-Analysis (PRISMA) statement (S1 Table).

### Search strategy

We conducted a systematic search in PubMed, Embase, Cochrane Library, and Web of Science databases for studies from the databases esteblished up to December 12, 2023 without restrictions on language. Keywords used were ("Dexmedetomidine") and ("Child" or "Adolescent" or "Pediatrics" or "Youth" or "Teen") and (intranasal) and (“postoperative delirium” or “postoperative agitation” or “emergenc delirium” or “emergenc agitation”). Search strategies were shown in S1 File.

### Eligibility criteria

Inclusion criteria were: 1) Children younger than 18 years. 2) American Society of Anesthesiologists (ASA) physical status I-III. 3) DEX group received intranasal DEX as premedication before anesthesia, and control group received normal saline. 4) The incidence of ED/ EA in DEX group and control group both could be converted into dichotomous variable. 5) Randomized controlled trial (RCT). Exclusion criteria were: 1) Other intervention that influenced ED/ EA was used. 2) Letter, abstract.

### Data extraction and bias assessment

Two researchers strictly screened the title and abstracts and full texts according to the eligibility criteria, then extracted characteristics of each study, such as authors, publication year, country, age, gender, and sample size. The primary outcome was the incidence of ED/ EA, which was assessed according to Pediatric Anesthesia Emergence Delirium score (PAED), or other delirium and agitation scale. ED/ EA was defined as PEAD score>10 or the equal level as the other grading scale. Secondary outcome included severe ED/ EA which was defined as PEAD score>14 or the equal level as the other grading scale, ED/ EA score, PACU pain score which was assessed according to 1–10 grading scale, parent’s satisfaction which was assesed according to 1–10 grading score, length of PACU stay, postoperative nausea and vomiting, breath holding. Cross-checked were conducted and a third researcher joined the discussion for consensus in cases of disagreement.

The quality assessment of included studies was independently conducted by two researchers using the Cochrane Risk of Bias tool Version 2.0 (RoB2), including five aspects: bias arising from the randomization domains, bias due to deviations from intended interventions, bias due to missing outcome data, bias in measurement of the outcome, bias in selection of the reported result. Any discrepancies were resolved through consultation with a third reviewer. To assess publication bias, funnel plots was performed and checked its symmetry if articles were more than nine.

### Data synthesis and analysis

All the data analyses were performed by R 4.3.0. Odd ratio (OR) with 95% CI was calculated for dichotomous variable, and standard mean difference (SMD) with 95% CI was calculated for continuous variable. Heterogeneity was determined using I^2^, a random-effects model was employed regardless of heterogeneity.

Network diagram was performed to show the direct and indirect comparison between different dosages, the size of the nodes represents the sample size of each dosage, and the thickness of the lines connecting the nodes indicated the number of studies directly compared between two dosage. When testing global consistency, P>0.05 indicates that there was no overall inconsistency, and the consistency model was used, otherwise non-consistency model was used. League tables were performed to summarize the different dosages of outcomes containing both direct and indirect comparisons. We calculated the probabilities of ranking in all different dosages to rank the effect of different dosages on outcomes.

## Results

A total of 159 articles were obtained from the databases, and 17 articles were retained after screening title and abstract. Finally, 15 RCTs with 1566 children were included in this network meta-analysis after full text screening [6, 7, 9–21]. Among these, 13 were from China, 1 was from Egypt, and 1 was from Australian. The investigated treatment arms included 0.5 μg/kg DEX, 1 μg/kg DEX, 1.5 μg/kg DEX, 2.0 μg/kg DEX, and normal saline.

**Fig 1:**
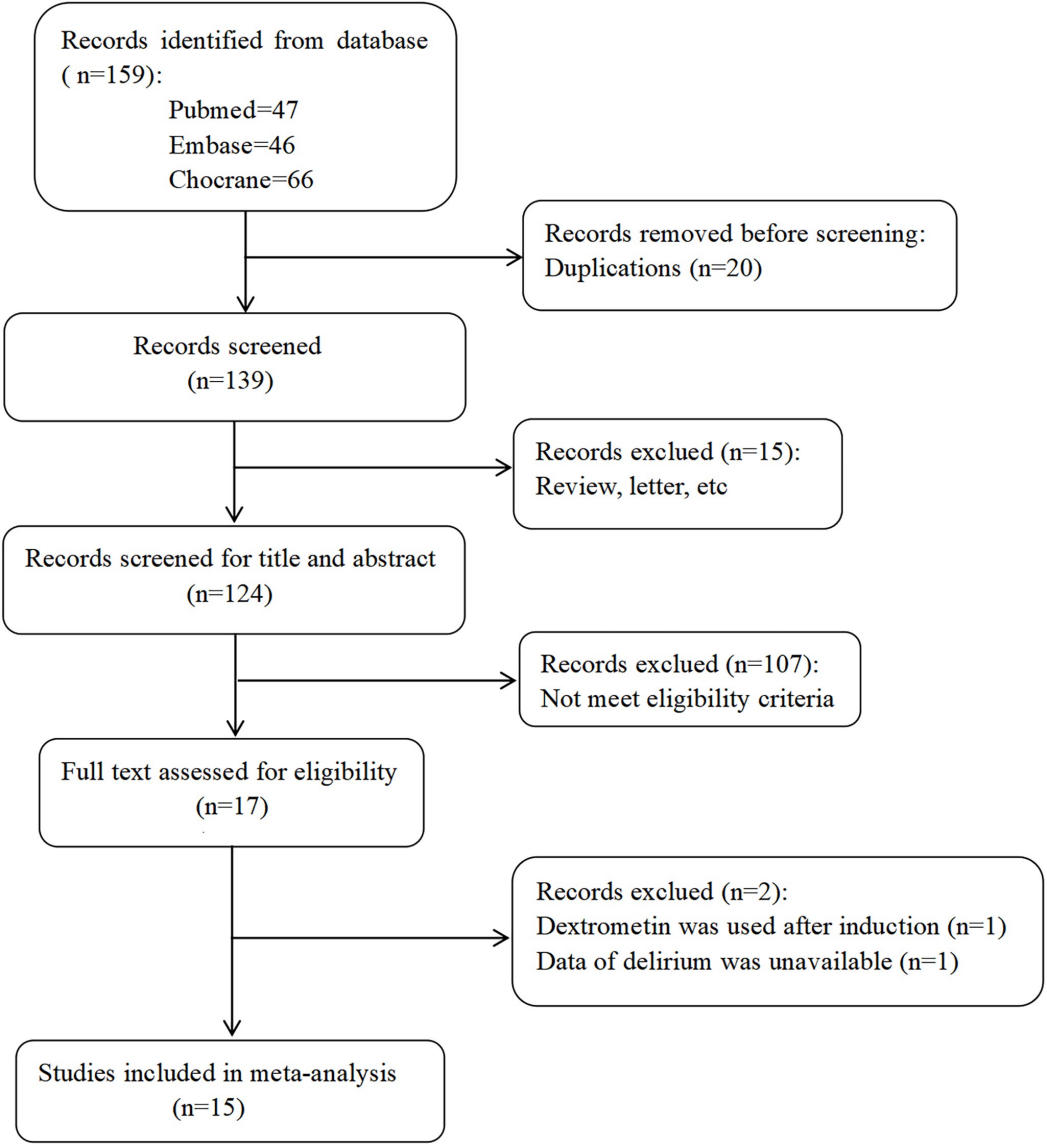
Flowchart of study screening process.

The PRISMA flowchart was shown in Fig 1, The characteristics of included studies was shown in Table 1.

**Table 1:** Characteristics of included studies on the different dosage of intranasal dexmedetomidine for anti-emergence delirium or agitation.

ID	Country size	Age ASA	Anesthesia	Elective surgery	Intervention and time	Delirium or agitation criteria
He H, 2023 [6]	China 90	3–7 I/II	GA: Sevo + Prop + rocuronium + Sufen; Sevo + Remifen BIS: 40–60; 35.5–37.0°C	Mouth dental rehabilitation	2 μg/kg DEX, 1 μg/kg DEX, Saline 30 min before surgery accompanied by one parent	PAED
Lei D, 2022 [7]	China 240	1–10 I/II	GA: Prop + nalbuphine + Sevo; Desflurane BIS: 40–60	Inguinal hernia repairs and hydrocoele	2 μg/kg DEX, 1.5 μg/kg DEX, 1 μg/kg DEX, 0.5 μg/kg DEX, Saline	Cole five-point scale
Shen F, 2022 [9]	China 248	0–12 I/II		Tonsillectomy and adenoidectomy	2 μg/kg DEX, Saline 30 to 60min before induction	PAED
Yao J, 2022 [10]	China 60	2–6 I	GA: Sevo + Sufen + etomidate + cisatracurium; dexamethasone + ketorolac	Tonsillectomy and/or adenoidectomy	1.0 μg/kg DEX, Saline 30 min before induction accompanied by one parent	PAED
Lee A, 2020 [11]	Australia 166	2–7 I/II	GA: Inhalational or intravenous	Day case procedures	2 μg/kg DEX, Saline 40 min before surgery accompanied by one parent	
Yao Y, 2020 [12]	China 103	2–6 I/II	GA: Sevo + Sufen +Prop; Sevo + NO Postoperative: proparacaine hydrochloride paracetamol	Unilateral strabismus surgery	2 μg/kg DEX, Saline 45 min before induction	PAED
Yan X, 2020 [13]	China 60	2–6 I	GA: Sevo	Monocular retinae cytoma	2 μg/kg DEX, Saline 30 min before induction	PAED
Zhang S, 2019 [14]	China 134	<16 II/III	GA: Midazolam + Fen + Prop	Interventional cardiac catheterisation	1.5 μg/kg DEX, Saline 30-45min before induction	Aonos four-point scale
Bi Y, 2019 [15]	China 40	6–48 months I/II	GA: Sevo BIS: 40–60	Tracheobronchial foreign body aspiration	1 μg/kg DEX, Saline 25 min before induction	Cole five-point scale
Gao L, 2018 [16]	China 60	2–9 I/II	GA: Sevo	Dental surgery	2 μg/kg DEX, Saline 30 min before induction	PAED
Li L, 2018 [17]	China 90	2–7 I/II	GA: cisatracurium + Prop; Prop + Remifen	Adenoidectomy	1 μg/kg DEX, 2 μg/kg DEX, Saline 25–45 min before induction	PAED
Ping L, 2018 [18]	China 60	3–7 I/II	GA	Tonsil and adenoidectomy, circumcision ringIncision, hernia	2 μg/kg DEX, Saline 30 min before induction, accompanied by one parent	PAED
Abdelaziz H, 2016 [19]	Egyptian 65	1–7 I/II	GA: Sevo + NO Postoperative: acetamiacetami	Strabismus surgery	1 μg/kg DEX Saline	PAED
Lin Y, 2016 [20]	China 90	1–8 I/II	GA: Sevo	Cataract surgeries	2 μg/kg DEX, 1 μg/kg DEX, Saline 45 min before induction	PAED
Yao Y, 2015 [21]	China	3–7 I/II	GA: Sevo + sufen + Prop; sevo + NO 36.8 ±0.4°C Postoperative: proparacaine hydrochloride paracetamol	Unilateral strabismus	2 μg/kg DEX, 1 μg/kg DEX Saline 45 min before induction	PAED

DEX: dexmedetomidine; GA: gerneral anesthesia; Sevo: sevoflurane; Prop: propofol; NO: nitrous oxide; Sufen: sufentanil; Remifen: Remifentanil; Fen: Fentanyl; PAED: Pediatric Anesthesia Emergence Delirium Scale

### Quality risk of bias and publication bias

There were no risk of bias in randomization and deviations from intended interventions in all 15 RCTs. The overall risk was “some concerns” mainly came from not reporting if all patient data was available, as shown in S1 Fig.

Funnel plots of publication bias in the 15 RCTs in all outcomes revealed general symmetry, indicated no signifcant publication bias, as shown in S2 Fig.

### Network meta-analysis

#### Primary outcome

**Fig 2:**
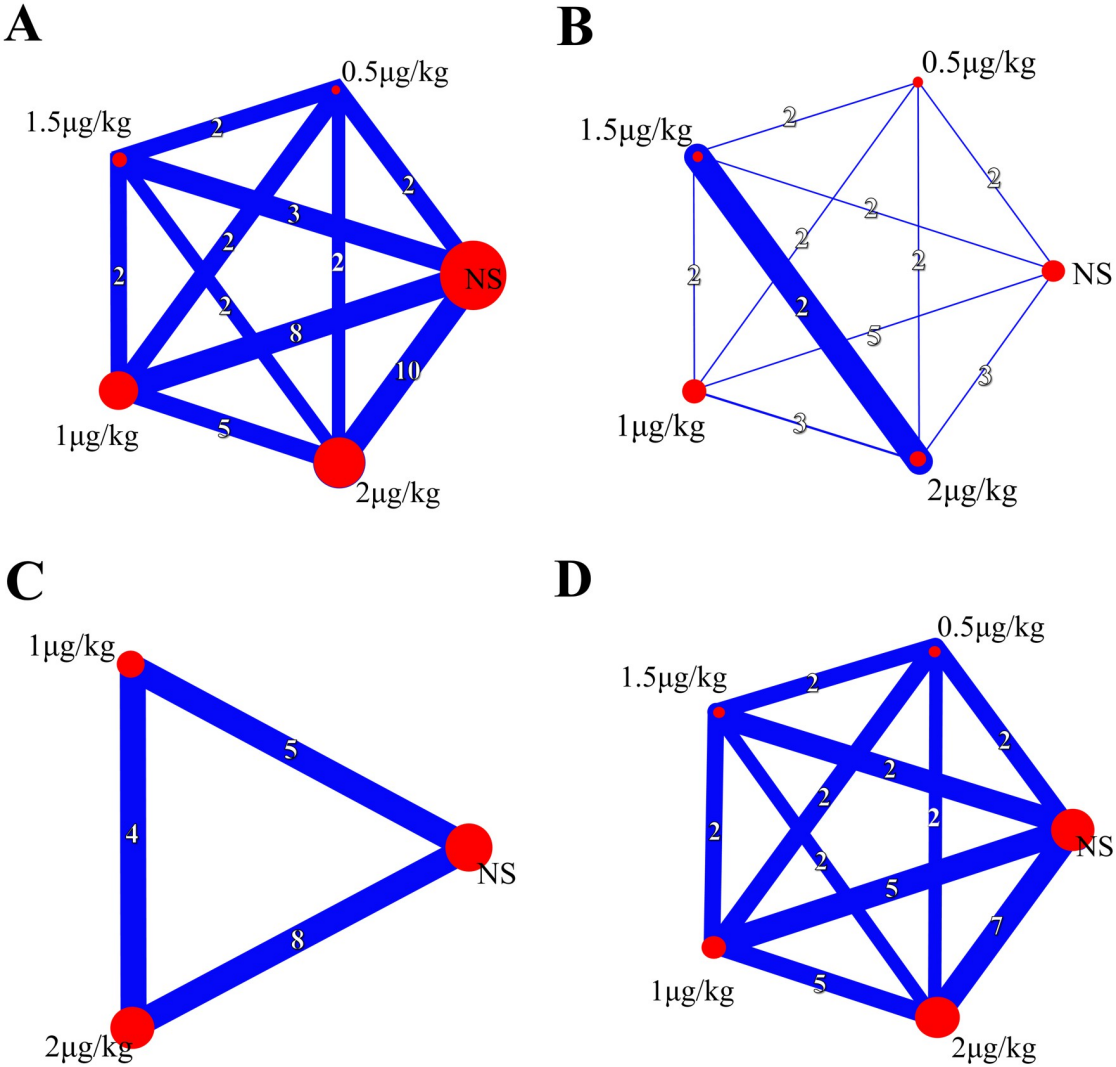
Network plot diagram for the effect of different intranasal dexmedetomidine dosage on outcomes. (A) the incidence of emergence delirium or emergence agitation; (B) the incidence of severe emergence delirium or emergence agitation; (C) Score of Pediatric Anesthesia Emergence Delirium Scale; (D) PACU pain VAS score. The thickness of edges and numbers located on the line represent the number of trials for each direct comparison.

**Fig 3:**
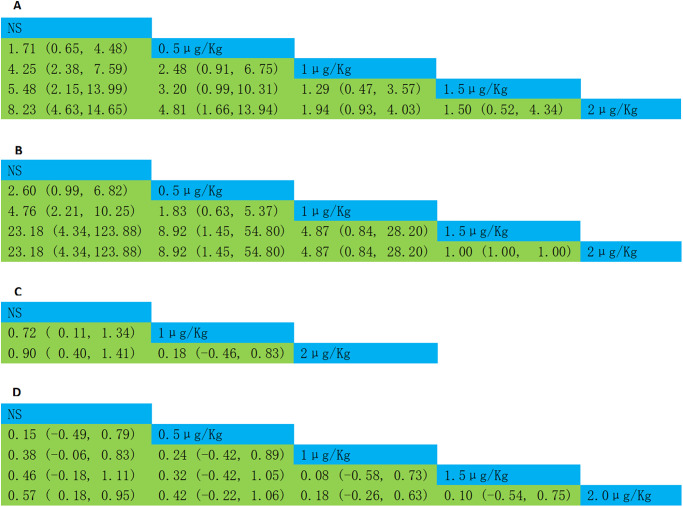
Network meta-analysis of different intranasal dexmedetomidine dosage on outcomes. (A) the incidence of emergence delirium or emergence agitation; (B) the incidence of severe emergence delirium or emergence agitation; (C) Score of Pediatric Anesthesia Emergence Delirium Scale; (D) PACU pain VAS score.

**Fig 4:**
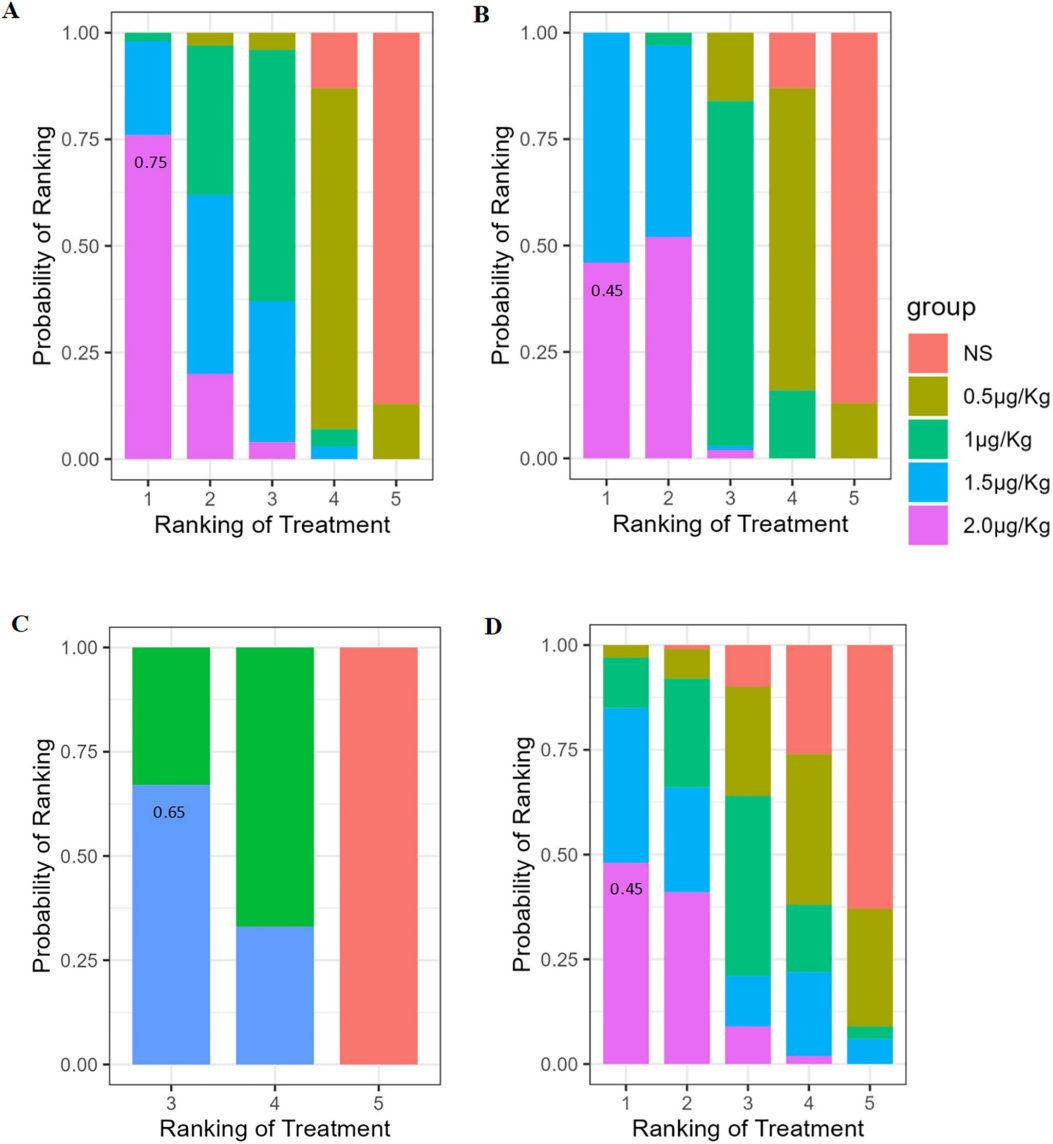
The probability of ranking plot for the effect of different intranasal dexmedetomidine dosage on outcomes. (A) the incidence of emergence delirium or emergence agitation; (B) the incidence of severe emergence delirium or emergence agitation; (C) Score of Pediatric Anesthesia Emergence Delirium Scale; (D) PACU pain VAS score.

*The incidence of ED/ EA*. 13 studies reported the incidence of ED/ EA, 2 μg/kg was the most reported dosage, as shown in network plot in Fig 2A. The consistency model showed no significant inconsistency showed between the direct comparisons and indirect comparisons, as shown in S3 Fig. The network meta analysis revealed that compared to 0.5 μg/kg (RR = 4.81, 95%CI = 1.66–13.94) and normal saline (RR = 8.23, 95%CI = 4.63–14.65), intranasal dexmedetomidine at the doses of 2 μg/kg significantly reduced the incidence of ED/ EA in children, as shown in Fig 3A. 2 μg/kg was the most effective dose in reducing the incidence of ED/ EA (Probability of rank = 0.75), as shown in Fig 4A.

#### Secondary outcome

*Severe ED/ EA*. five studies reported the incidence of severe ED/ EA, 1 μg/kg was the most reported dosage, as shown in network plot in Fig 2B. No significant inconsistency showed between the comparisons was shown in S3 Fig. The network meta analysis revealed that compared to 0.5 μg/kg (RR = 8.92, 95%CI = 1.45–45.80) and normal saline (RR = 23.18, 95%CI = 4.34–123.88), intranasal dexmedetomidine at the doses of 2 μg/kg significantly reduced the incidence of ED/ EA in children, as shown in Fig 3B. 2 μg/kg was the most effective dose in reducing the incidence of ED/ EA (Probability of rank = 0.45), as shown in Fig 4B.

*ED/ EA score*. nine studies reported the ED/ EA score, 2 μg/kg was the most reported dosage, as shown in network plot in Fig 2C. No significant inconsistency showed between the direct comparisons and indirect comparisons, as shown in S3 Fig. The network meta analysis revealed that compared to 1 μg/kg (RR = 0.18, 95%CI = -0.46–0.83), intranasal dexmedetomidine at the doses of 2 μg/kg significantly reduced the incidence of ED/ EA, as shown in Fig 3C. 2 μg/kg was the most effective dose in reducing the incidence of ED/ EA (Probability of rank = 0.65), as shown in Fig 4C.

*PACU pain score*. six studies reported the PACU pain score, 2 μg/kg was the most reported dosage, as shown in network plot in Fig 2D. No significant inconsistency showed between the direct comparisons and indirect comparisons, as shown in S3 Fig. The network meta analysis revealed that intranasal dexmedetomidine at 2 μg/kg significantly reduced pain compared to 1 μg/kg (RR = 0.18, 95%CI = -0.26–0.63), 1.5 μg/kg (RR = 0.10, 95%CI = -0.54–0.75), and normal saline (RR = 0.57, 95%CI = 0.18–0.95), as shown in Fig 3D. 2 μg/kg was most effective dose in reducing the incidence of ED/ EA (Probability of rank = 0.45), as shown in Fig 4D.

There were four studies each reporting parent’s satisfaction, length of PACU stay, postoperative nausea and vomiting, breath holding. Since limited data, we did not pool these outcomes, as shown in S2 File.

## Discussion

Our network meta-analysis found that 2μg/kg preoperative intranasal dexmedetomidine was the most effective doses in reducing the incidence of postoperative ED/ severity of EA, ED/ EA, and ED/ EA score compared with 0.5μg/kg, 1μg/kg, 1.5μg/kg, and normal saline in children. In addition, 2μg/kg was also the most effective in relieving pain in PACU. These findings were helpful for the guidance of clinical medication.

A lot of research supported dexmedetomidine can prevent postoperative delirium. Jen et al. [22] reported that oral dexmedetomidine was effective in reducing sudden agitation in pediatric patients. Dexmedetomidine might prevent ED/ EA in children by promoting NLRP3 inflammasome degradation [23] and reducing hippocampal brain inflammation [24] through the autophagy-ubiquitin pathway, thereby protecting nerves and improving cognitive dysfunction.

At present, only one meta-analysis by Na et al. [25] reported the relationship between intranasal dexmedetomidine and ED/ EA, and concluded that intranasal dexmedetomidine reduced ED/ EA after general anesthesia from 33.2% to 13.6%. This was similar to our findings. However, their study included few studies, mixed drug types in control group, including midazolam, clonidine, ketamine, etc., and no subgroup analysis of different dexmedetomidine dosage, making it unreliability in clinical application. Another meta analysis by Zhou et al. [26] showed that the ED90 of intravenous DEX for preventing EA in pediatric patients undergoing dental surgery with sevoflurane anesthesia was 0.74 μg/kg/h. However, their administration route was intravenous. Our network meta-analysis confirmed the anti-ED/ EA efficacy of dexmedetomidine compared to saline, moreover, we concluded that 2 μg/kg was the optimal dosage via aspects of the incidence, severity, and score of ED/ EA.

We revealed intranasal dexmedetomidine reduced pain in PACU, and 2 μg/kg was the optimal dosage. Fu et al. [27] and Tang et al. [28] showed that intravenous dexmedetomidine reduced postoperative pain in children, which was consistent with ours.

There are some limitations in our study. The number of included studies were small and we did not included non-randomized studies not only for they had lots of confounding factors, but there was no non-randomised study(observational or cohort studies) reporting intranasal dexmedetomidine preventing pediatric patients’ ED/ EA as well. We did not compare the anti-ED/EA effects of different pathways of dexmedetomidine, such as intravenous, oral route. These are needed future studies.

## Conclusions

2 μg/kg was the optimal dosage for preventing postoperative ED/ EA and pain. These findings might provided guidance of clinical medication. However, more large-sample, multicenter clinical studies are warranted to further enhance the conclusions.

## Supporting information

S1 Table PRISMA_2020_checklist. (DOCX)

S1 Fig Risk of bias of included studies. (JPG)

S2 Fig Funnel plot. (TIF)

S3 Fig Node-splitting_forest. (TIF)

S1 File Search strategy for network meta analysis. (DOCX)

S2 File Data of included studies. (DOCX)
