# Supplementary figures and images for: The effect of different dosage of intranasal dexmedetomidine on preventing emergence delirium or agitation in children: A network meta-analysis of randomized controlled trials
Source: PLoS One. 2024 Sep 6;19(9):e0304796. doi: 10.1371/journal.pone.0304796 (PMC11379244; doi:10.1371/journal.pone.0304796)

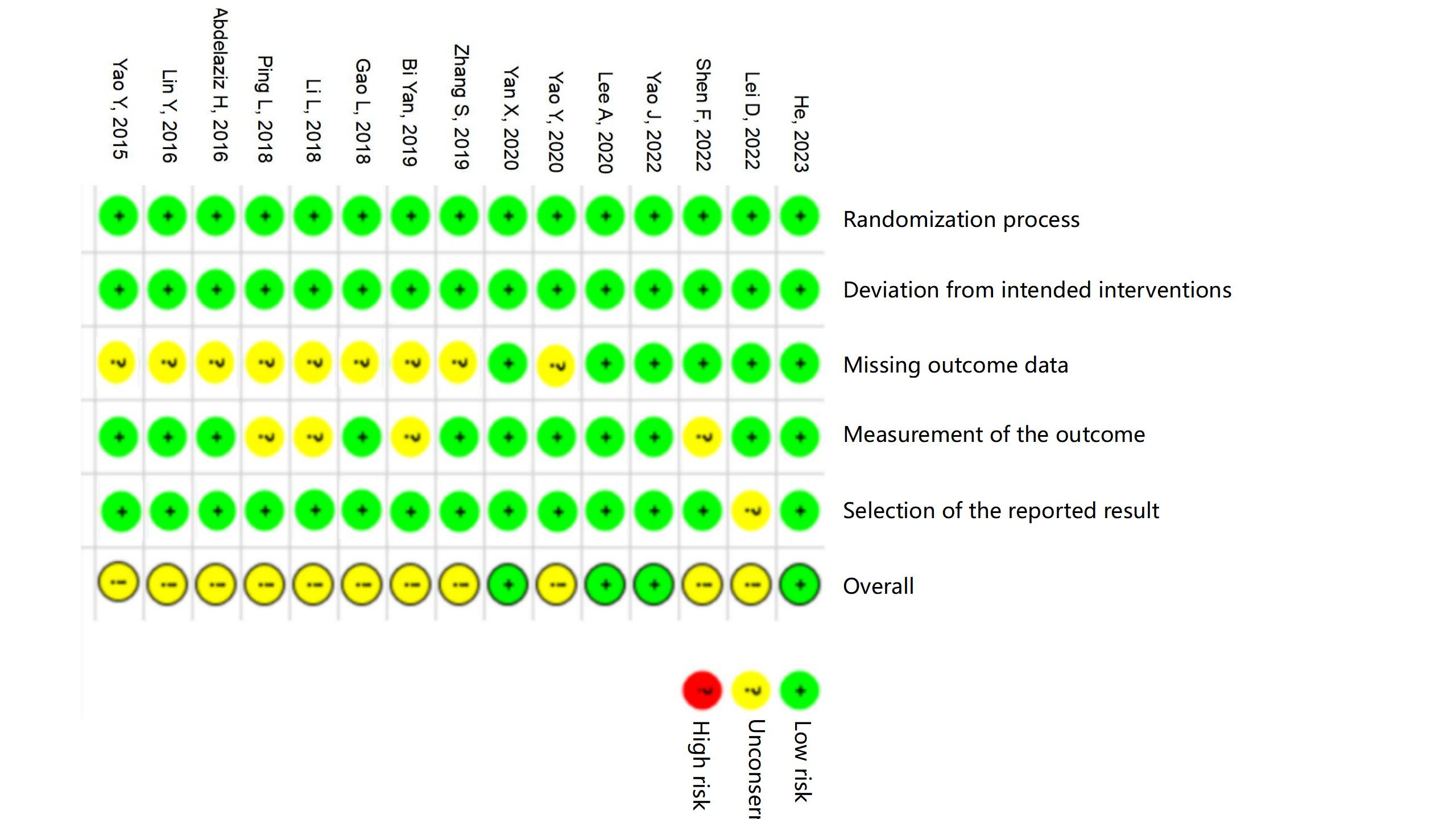

Supplement: S1 Fig — (JPG) [file pone.0304796.s002.jpg]

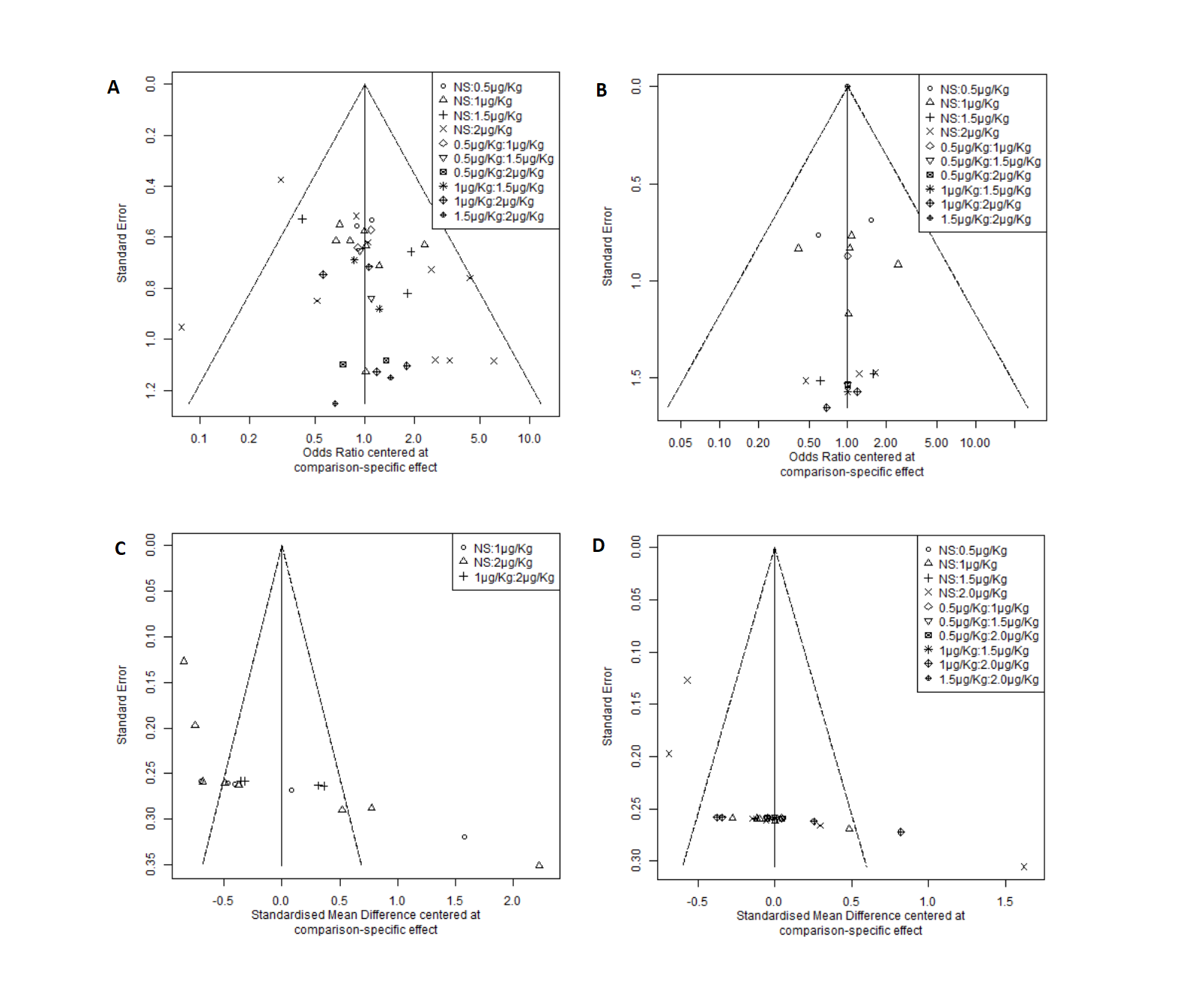

Supplement: S2 Fig — (TIF) [file pone.0304796.s003.tif]

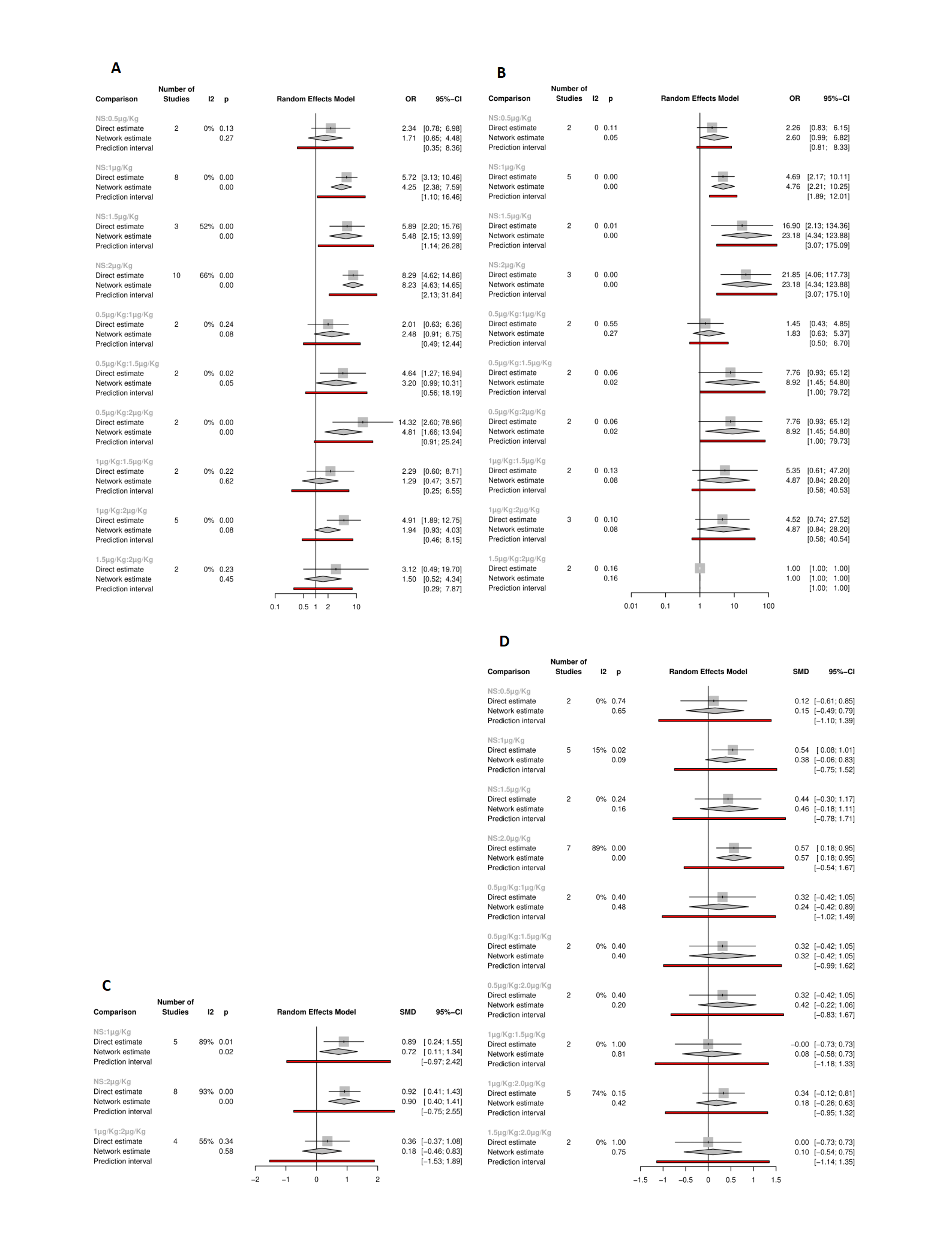

Supplement: S3 Fig — (TIF) [file pone.0304796.s004.tif]
