# Supplementary material for: The effect of different dosage of intranasal dexmedetomidine on preventing emergence delirium or agitation in children: A network meta-analysis of randomized controlled trials
Source: PLoS One. 2024 Sep 6;19(9):e0304796. doi: 10.1371/journal.pone.0304796 (PMC11379244; doi:10.1371/journal.pone.0304796)
Supplement: S1 File — (DOCX) [file pone.0304796.s005.docx]

#1 nasal OR intranasal

#2 dexmedetomidine[Title/Abstract]

#3 (((children) OR (Pediatric)) OR (c)) OR (teenager)

#4 "emergence delirium" OR "emergence agitation" OR "postoperative delirium" OR "postoperative agitation"

#5 #1 and #2 and #3 and #4 47

Chocrane:66

Embase:46
