# Supplementary material for: The effect of different dosage of intranasal dexmedetomidine on preventing emergence delirium or agitation in children: A network meta-analysis of randomized controlled trials
Source: PLoS One. 2024 Sep 6;19(9):e0304796. doi: 10.1371/journal.pone.0304796 (PMC11379244; doi:10.1371/journal.pone.0304796)
Supplement: S2 File — (DOCX) [file pone.0304796.s006.docx]

| Study id | **ID** | **dosage/**  **size** | **ED/ EA** | **severe ED/ EA** | **ED/ EA score** | **PACU pain** |
| --- | --- | --- | --- | --- | --- | --- |
| 1 | He H, 2023 | 1ug/ 2ug/ C =30:30:30 | 11  3  21 | 1  0 11 | 8.39±4.28 5±3.11 13±6.23 | 3.93±1.71 2±1.56 5.64±1.71 |
| 2 | Lei D, 2022(1-3y) | 0.5ug/ 1ug/ 1.5ug/ 2ug/ C =30:30:30:30:30 | 12 7 4 1 19 | 3 2 0 0 9 |  | 2.21±2.34 1.36±2.34 1.36±2.34 1.36±2.34 2.36±2.34 |
| 3 | Lei D, 2022(3-10y) | 0.5ug/ 1ug/ 1.5ug/ 2ug/ C =30:30:30:30:30 | 8 5 2 1 13 | 3 2 0 0 4 |  | 2±1.56 1.36±2.34 1.36±2.34 1.36±2.34 2.43±3.11 |
| 4 | Shen F, 2022 | 2ug/ C =124:125 | 12  27 |  | 5±4.67 5.36±5.45 | 1.3±1.5 1.3±1.5 |
| 15 | Yao J, 2022 | 1ug/ C =30:30 |  |  | 5.1 ± 2.7 6.7 ± 3.8 |  |
| 5 | Lee A, 2020 | 1ug/ C =30:30 | 1 5 |  |  |  |
| 6 | Yao Y, 2020 | 2ug/ C =52:51 | 6  25 |  | 7.43±9.34 9.14±10.90 | 0.73±0.57 0.65±0.76 |
| 7 | Yan X, 2020 | 2ug/ C =30:30 | 5 19 |  | 6.72±7.78 11.64±10.12 |  |
| 8 | Zhang S, 2019 | 1.5ug/ C =67:67 | 6 13 |  |  |  |
| 9 | Yanmei B, 2019 | 1ug/ C =20:20 | 5 14 | 1 4 |  |  |
| 10 | Gao L, 2018 | 2ug/ C =30:30 | 3 2 |  |  |  |
| 16 | Li L, 2018 | 1ug/ 2ug/ C =30:30:30 |  |  | 7.36±2.34 7.36±2.34 8.36±2.34 | 3±1.56 2.14±0.78 3.39±1.79 |
| 11 | Li P, 2018 | 2ug/ C =30:30 | 2 7 |  | 5.71±1.56 8.07±1.71 |  |
| 12 | Abdelaziz H, 2016 | 1ug/ C =35:35 | 4 15 | 2 8 |  |  |
| 13 | Yiquan L, 2016 | 1ug/ 2ug/ C =30:30:30 | 7 3 24 |  | 8.85±15.57 8.28±16.57 11.94±14.79 | 3.5±7.01 3.85±7.78 9.21±14.79 |
| 14 | Yao Y，2015 | 1ug/ 2ug/ C =30:30:29 | 5 1 14 |  | 4.39±1.79 3 ±1.56 9.53±2.72 |  |
